# Mental Imagery to Enhance Procedural Skills in Peripheral Venous Catheterization: A Randomized Simulation Study in Medical Students

**DOI:** 10.5334/pme.2069

**Published:** 2026-02-18

**Authors:** Nicolas Boulet, Hugo Nairv, Myriam Mezzarobba, Laysa Saadi, Denis Morin, Claire Roger

**Affiliations:** 1 UR-UM103 IMAGINE, University of Montpellier, Division of Anesthesia Critical Care, Pain and Emergency Medicine, Nîmes University Hospital, Montpellier, France; 2 Department of Biostatistics, Epidemiology, Public Health and Innovation in Methodology (BESPIM), CHU Nimes, IDESP, INSERM, University of Montpellier, Nîmes, France; 3 Pediatric Nephrology, CHU Montpellier, SORARE reference center, Montpellier, France

## Abstract

**Introduction:**

Procedural skill acquisition is fundamental to medical education, yet training opportunities are increasingly constrained. Mental imagery, a cognitive rehearsal method shown to enhance psychomotor performance in various domains, has gained attention in medical training as a low-cost, safe tool to improve technical proficiency. Evidence remains limited and heterogeneous. Specifically, its application to peripheral venous catheter (PVC) insertion, a frequent and essential clinical procedure, has scarcely been studied.

**Methods:**

In this single-center, open-label, randomized controlled simulation trial, fifth-year medical students from Nîmes University Medical School were randomized to either a mental imagery or control group prior to a simulated PVC insertion on a mannequin. Primary outcome was uncomplicated first-puncture success rate. Secondary outcomes included variations in adapted Mental Imagery Questionnaire (MIQ) score, number of punctures, and procedural complications.

**Results:**

Sixty-four students were included (33 in the mental imagery group, 31 in the control group). Baseline characteristics were similar, although the control group had greater prior experience with PVC insertion. The first-attempt success rate was significantly higher in the mental imagery group (76% versus 52%, p = 0.04). Complications occurred less frequently in the mental imagery group (0% versus 12.9%, p < 0.05). The median improvement in the adapted MIQ score was significantly greater in the mental imagery group (+17 [+12, +22] versus +11 [+8, +18]; p = 0.004), indicating enhanced mental readiness and procedural confidence.

**Conclusion:**

Mental imagery is an effective, safe, low-cost strategy that significantly enhances simulation-based procedural performance and learner confidence in PVC insertion.

## Introduction

The acquisition of procedural skills is a cornerstone of medical education, essential for future clinical practice. With increasing pressures on clinical training time and a growing emphasis on patient safety, the exploration of supplementary and innovative pedagogical methods to enhance procedural competence is essential.

Mental imagery, defined as the cognitive rehearsal of a task in the absence of actual physical execution, has been established as a valuable technique for enhancing performance across various domains requiring complex psychomotor skills, such as sports [1,2,3]. Through the activation of neural networks similar to those involved in physical practice, mental imagery facilitates the consolidation of procedural knowledge, enhances cognitive and motor performance, and offers significant potential for optimizing the acquisition and refinement of technical skills in medicine and surgery [4,5]. Mental imagery is also recognized for its cost-effectiveness and safety [6], as it does not necessitate expensive equipment.

In this context, mental imagery has garnered increasing attention within medical education [7]. In surgical practice, mental rehearsal is often used intuitively by experienced surgeons to prepare for procedures [8]. Several prospectives studies suggest that mental rehearsal can positively impact the acquisition and retention of surgical or procedural skills [5,6,9,10,11,12]. Systematic reviews have further indicated that mental practice may contribute to technical performance enhancement, stress management, and learner confidence [13,14].

Beyond individual technical skills, mental imagery could enhance collective team performance, particularly in high-risk situations. For instance, a randomized simulation study demonstrated that mental imagery could improve teamwork competencies more effectively than traditional simulation-based instruction in the context of emergency trauma care [15].

However, the overall body of evidence remains heterogeneous, and the extent to which mental imagery can be systematically integrated into routine medical training warrants further investigation [16].

The acquisition of procedural skills constitutes a core element of the education of healthcare professionals, including those in medical, surgical, and nursing schools. Given that many technical procedures inherently expose patients to risks of discomfort or harm, the development of modern, safer educational strategies has become essential. Among these skills, peripheral venous catheter (PVC) insertion represents one of the most frequently performed procedures, playing a critical role in the administration of intravenous therapies across diverse clinical settings. To date, the specific application of mental imagery to the learning of PVC insertion has been the subject of limited investigation, with a single study reporting promising outcomes, thereby highlighting the need for further research in this area [10].

The aim of this study was to evaluate the impact of pre-procedural mental imagery on procedural performance during PVC insertion in a mannequin-based simulation among fifth-year medical students.

## Methods

### Study Design

We conducted a monocentric randomized controlled open-label study, at the Nîmes University Hospital, France.

According to French law [17], ethical approval for this study was provided by the local Ethical Committee (approval number 24.11.07), and registered at clinicaltrials.gov (NCT06932822).

### Population

Inclusion criteria: all 96 fifth-year medical students from the Nimes medical school were invited to participate in a simulation PVC procedure training day at the university, during the 2024–2025 academic year. All participants were eligible for inclusion in the study. Participation in the study was voluntary and uncompensated.

### Randomization

Participants were randomly assigned into two groups using a computer-generated four-size blocks randomization: the mental imagery group, and the control group.

By design, the participants were aware of their assigned group.

### Interventions

Prior to the workshop, participants were required to watch a tutorial video detailing the PVC procedure. Then, the procedural workshop consisted of attempting to insert a catheter into a low-fidelity mannequin arm, without any additional assistance or further instruction.

Prior to the procedure, the mental imagery group underwent a 2-minute session of mental imagery. In this session, supervisors read aloud a standardized script on PVC insertion to participants with their eyes closed (Supplemental Figure S1). The script was adapted from Collet et al. [10] and designed to promote an internal perspective (first-person view) while emphasizing kinesthetic sensations, encouraging participants to mentally simulate the procedure as if they were physically performing it.

### Data Collection

Demographic data of the participants were collected, including: age, sex, right or left-handed, previous experience in PVC insertion, site of PVC insertion, first puncture success rate, number of punctures, arterial puncture, failure, and non-functioning perfusion.

Previous experience in PVC insertion was defined in 5 levels, according to the previous number of PVC already inserted by the student: none, one, between 2 and 5, between 5 and 10, and more than 10.

Failure was defined as an absence of success after 3 skin punctures.

The perceived quality of mental imagery was assessed using the adapted Mental Imagery Questionnaire (MIQ). This instrument, originally derived from the Movement Imagery Questionnaire [18,19], was previously adapted and validated for surgical procedures [5,20]. This questionnaire has been adapted to specifically assess PVC insertion (Supplementary Figure S2). The adapted MIQ consists of 8 items capturing the quality and vividness of participants’ mental imagery related to the PVC insertion before and after the procedural workshop, by assessing different key dimensions: confidence in procedure execution, procedural knowledge, ease of generating mental imagery (“seeing”), ease of generating kinesthetic imagery (“feeling”), overall mental readiness, and the perceived usefulness of the procedural workshop. Each question was rated using a 7-point Likert scale. Both groups were asked to complete the adapted MIQ before and after the PVC procedural workshop.

All data collected were anonymized using a unique identification number.

### Outcomes

The primary outcome was uncomplicated first-puncture success rate, defined as successful cannulation on the first skin puncture resulting in functional perfusion, with no associated complications (arterial puncture or hematoma).

The secondary outcomes were: the absolute value and the variation in the adapted MIQ score (calculated as the difference between the adapted MIQ score after and before the procedural workshop), before and after the procedural workshop; the number of skin punctures; and the composite outcome of complications (arterial puncture, failure, non-functioning perfusion).

### Statistical Analysis

All analyses were conducted using R Version 4.4.2 (R Foundation, Vienna, Austria) [21].

Continuous data are reported as medians with interquartile ranges [IQR], and categorical data are reported as numbers and percentages.

Categorical variables were compared using the Chi-squared test (uncomplicated first-puncture success rate) or Fisher’s exact test (complications), as appropriate. To quantify the magnitude of the effect for the primary outcome, we estimated the Odds Ratio (OR) with its 95% Confidence Interval (95% CI) using a univariate logistic regression model. To account for baseline imbalances in prior proficiency, a multivariable logistic regression analysis was performed on the primary outcome. The model included the randomized group and prior PVC experience, categorized as “Novice” (0 to 1 prior procedure) versus “Non-Novice” (≥ 2 prior procedures) to ensure adequate subgroup sizes. An interaction term was tested in a separate model to evaluate if the intervention effect differed by experience level.

Comparisons of continuous variables (MIQ scores, number of punctures) were performed using the non-parametric Wilcoxon rank-sum test (Mann-Whitney U test) for inter-group comparisons. Within-group changes (before versus after) were assessed using the Wilcoxon signed-rank test. The magnitude of the effect was reported as the median difference with its 95% CI. To ensure the psychometric quality of the adapted instrument in our population, we assessed its internal consistency using Cronbach’s alpha coefficient at both pre- and post-test time points.

A p-value less than 0.05 was considered significant, without correction for multiple testing.

## Results

**Figure 1:**
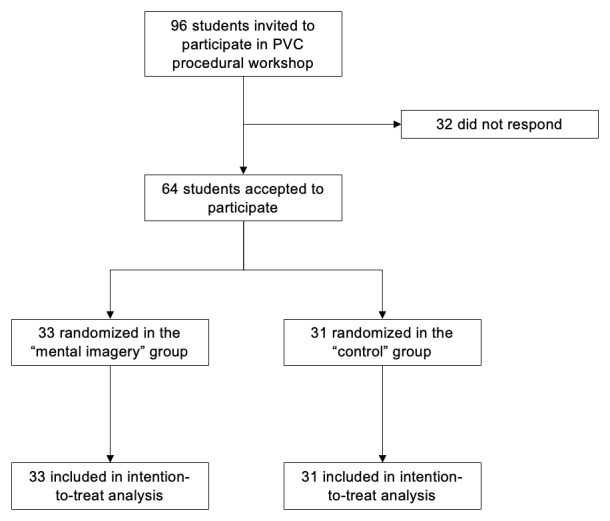
Study Flow Chart. PVC: peripheral venous catheter.

During the 2024–2025 academic year, we included a total of 64 fifth-year medical students from the Nîmes Faculty of medicine (Figure 1). 32 students were not available to participate. 33 students were allocated to the mental imagery arm and 31 to the control arm. In the mental imagery group, nearly half of the participants (48%) had no prior experience, compared to 29% in the control group (Table 1). Conversely, a greater proportion of participants in the control group reported intermediate (2–5 procedures: 29%) or higher levels of experience (>10 procedures: 9.7%) than in the mental imagery group (18% and 6.1%, respectively). These differences suggest that the control group was overall more experienced in PVC insertion. No other differences were observed at baseline in the demographic characteristics of the two groups (Table 1).

**Table 1:** Characteristics of the student population.


CHARACTERISTIC	MENTAL IMAGERY N = 33	CONTROL N = 31	OVERALL N = 64

Age			

Median [Q1, Q3]	22 [21, 23]	23 [22, 23]	22 [22, 23]

Sex			

Male	10 (30%)	11 (35%)	21 (33%)

Female	23 (70%)	20 (65%)	43 (67%)

Right-Handed			

No	4 (12%)	7 (23%)	11 (17%)

Yes	29 (88%)	24 (77%)	53 (83%)

Previous experience in PVC insertion			

0	16 (48%)	9 (29%)	25 (39%)

1	8 (24%)	7 (23%)	15 (23%)

2–5	6 (18%)	9 (29%)	15 (23%)

6–10	1 (3.0%)	3 (9.7%)	4 (6.3%)

> 10	2 (6.1%)	3 (9.7%)	5 (7.8%)

Insertion site			

Forearm	17 (52%)	17 (55%)	34 (53%)

Arm	11 (33%)	11 (35%)	22 (34%)

Elbow joint	5 (15%)	3 (9.7%)	8 (13%)


**Figure 2:**
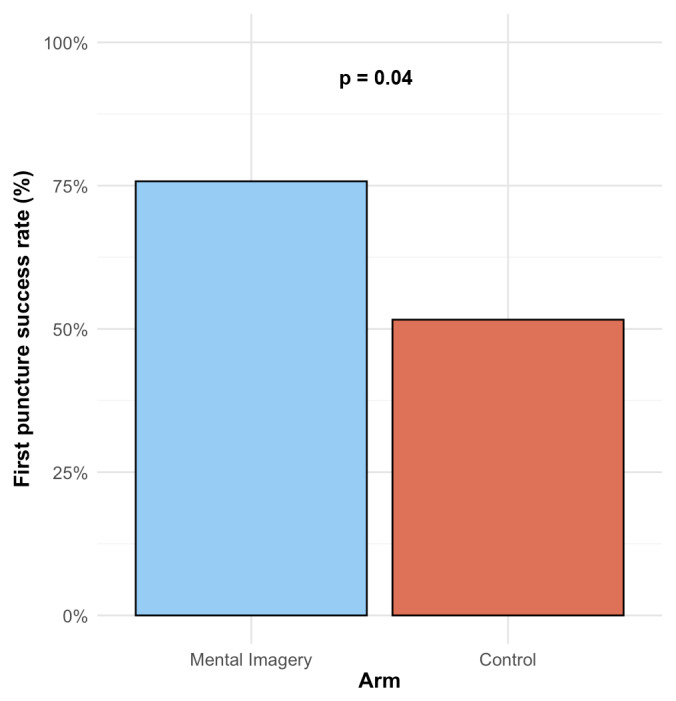
Uncomplicated first-puncture success rate. The uncomplicated first-puncture success rate was significantly higher in the mental imagery group (n = 25/33, 76%) compared to the control group (n = 16/31, 52%).

**Figure 3:**
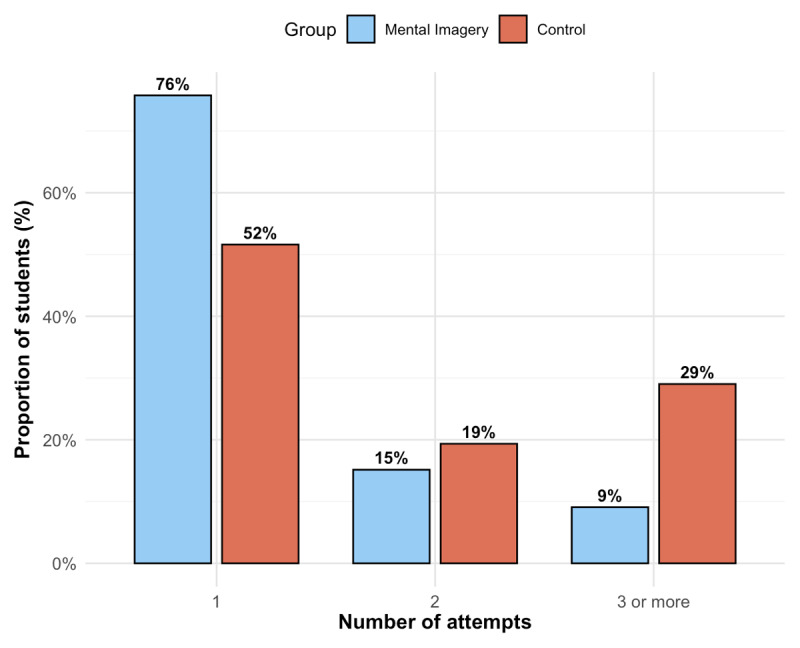
Distribution of the number of punctures by group. The bar chart displays the proportion of students requiring 1, 2, or ≥ 3 punctures to achieve success.

**Figure 4:**
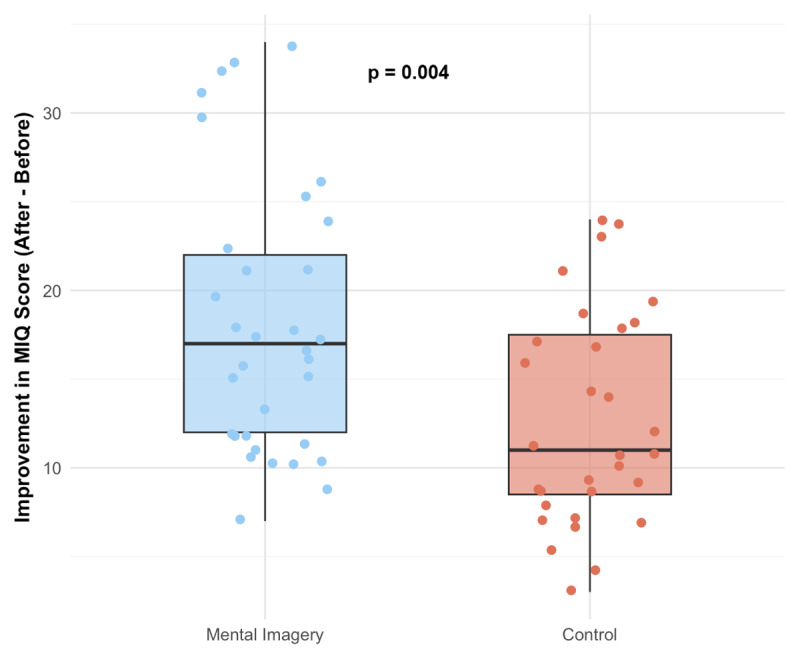
Variation in the adapted MIQ score. Boxplots represent the median and interquartile range (IQR) of the score improvement (Delta MIQ) in each group. Individual data points are overlaid. The increase in MIQ score was significantly higher in the mental imagery group compared to the control group (median = +17 [+12, +22], versus +11 [+8, +18], respectively).

The uncomplicated first-puncture success rate was statistically higher in the mental imagery versus control group, occurring in 25 (76%) versus 16 (52%), respectively (OR = 2.93 [1.03–8.81]; p = 0.04) (Figure 2). In the multivariable analysis adjusting for baseline experience (“Novice” versus “Non-Novice”), the mental imagery group remained significantly associated with a higher uncomplicated first-puncture success rate (adjusted OR = 3.02 [1.04 to 9.40]; p = 0.047). In this model, being experienced was not independently associated with higher success (p = 0.80). Furthermore, no significant interaction was found between the intervention and experience level (p for interaction = 0.25). The median number of punctures was significantly lower in the mental imagery compared to the control group (1 [1–1] versus 1 [1–3], respectively; p = 0.03) (Figure 3). The composite outcome of complications was statistically lower in the mental imagery versus control group, occurring in 0 (0%) versus 4 (12.9%), respectively (p < 0.05). Non-functioning perfusion occurred in 0 (0%) and 3 (9.7%) cases in the mental imagery versus control group, respectively (p = 0.11). Failure occurred in 0 (0%) and 1 (3.2%) case in the mental imagery versus control group, respectively (p = 0.48). No arterial puncture occurred in either group. The adapted MIQ score before the procedural workshop was not statistically different between the control and the mental imagery groups (34 [27–37] versus 32 [22–35], respectively; p = 0.20). Both groups showed a significant improvement in their MIQ scores after the workshop (p < 0.001 for both). However, the magnitude of this improvement was significantly greater in the mental imagery group. The median increase in adapted MIQ score was significantly greater in the mental imagery group compared to the control group (+17 [12–22] versus +11 [8–18], respectively; p = 0.004). The estimated median difference between groups was +5 [2 to 8] (Figure 4). The adapted MIQ demonstrated excellent internal reliability in our sample, with a Cronbach’s alpha of 0.90 at baseline and 0.88 after the workshop. Detailed results of the adapted MIQ score were reported in Supplementary Table S1.

## Discussion

This monocentric, randomized controlled simulation study demonstrates that a single mental imagery session performed prior to PVC procedure significantly improved uncomplicated first-puncture success rates among medical students. Moreover, this intervention was associated with a significant reduction in complications and enhanced the perceived quality of mental imagery, as evidenced by a significantly greater increase in adapted MIQ scores in the mental imagery group. This finding suggests that the quality of mental imagery may be a key mediator of the intervention’s effectiveness. Interestingly, despite a lower level of prior experience with PVC insertion in the mental imagery group, participants in this group achieved better outcomes, suggesting that mental imagery may compensate for limited practical experience and enhance procedural performance even among novices.

Non-technical interventions, such as cognitive training and mental imagery, are increasingly recognized as effective strategies to improve procedural performance and patient safety in medical education [7]. However, evidence regarding their specific impact on technical performance acquisition, particularly in the context of simulation-based learning, remains limited.

The relevance of mental imagery in procedural learning is further supported by the meta-analysis conducted by Sattelmayer et al. [14], which identified mental practice as one of the most promising motor learning strategies in medical and surgical education. Although individual randomized trials in this field often lack statistical power, this meta-analysis provides valuable evidence for the efficacy of such interventions.

Our findings complement those of Collet et al. [10], who investigated the effect of mental imagery on the learning curve for PVC insertion over multiples training sessions. Interestingly, their study also employed the MIQ scale to assess the quality of mental imagery, similar to our approach. In their study, medical students in the mental imagery group gained technical skills after four sessions, whereas those in the control group required five sessions to reach comparable performance. However, the beneficial effects reported by Collet et al. appeared to be confined primarily to the early phase of skill acquisition, suggesting that mental imagery may have the greatest impact during the initial learning stages rather than on later refinement of performance. A critical limitation of their study is the choice of procedural speed as the main outcome to validate learning curve progression. Although faster execution can reflect a certain level of technical expertise, speed alone is not necessarily a reliable surrogate for procedural quality or patient-centered outcomes.

Our study presents several strengths that reinforce the validity and relevance of its findings. First, the randomized controlled design enhances both the internal and external validity of the findings. Second, in contrast to previous research focusing primarily on learning procedures or skill acquisition [13], our work is, to our knowledge, the first simulation trial to specifically assess the direct impact of mental imagery on procedural performance, defined by clinically meaningful safety outcomes, regardless of the learner’s initial skill level. By selecting uncomplicated first-puncture success rate and complication incidence as primary and secondary outcomes, we deliberately emphasized performance quality rather than learning progression alone. This distinction is particularly relevant considering the umbrella review conducted by Snelgrove et al. [13], which highlighted a major gap in the existing body of evidence on motor learning interventions in health professions education. The authors demonstrated that most previous studies have focused on the early phases of skill acquisition, often evaluating knowledge acquisition or behavioral change, while rarely addressing higher-level outcomes such as actual performance improvement or patient benefit. This observation aligns with Kirkpatrick’s model of educational outcomes, in which the ultimate levels (Level 3: behavior, and Level 4: results) remain the least explored in procedural skill research [22]. By focusing on procedural success and complication rates, our study directly addresses these higher levels of Kirkpatrick’s framework, thereby offering evidence of the potential clinical relevance of mental imagery beyond its effect on learning dynamics.

Several limitations of this study should be acknowledged. First, due to the nature of the study design, the study was conducted in an open-label setting, and both investigators and participants were aware of the group allocation. To minimize the risk of assessment bias, we selected objective and pragmatic primary and secondary outcomes. However, we acknowledge that the participants’ awareness of the intervention may have introduced a performance bias (Hawthorne effect), leading to increased focus in the mental imagery group. Second, the intervention group received a dedicated guided mental imagery session immediately prior to the procedure. While all participants viewed a standardized instructional video on PVC insertion in the days preceding the workshop to equalize baseline knowledge, the control group did not perform an attention-matched neutral task immediately before the simulation. Therefore, we cannot exclude that the benefits observed were partly influenced by an enhanced immediate focus in the intervention group. Third, the voluntary nature of participation might induce a selection bias favoring motivated students. However, this does not affect the internal comparison between groups, and the study sample remains representative of the target audience of active learners willing to engage in simulation training. Finally, procedural performance was assessed at a single time point, immediately following the intervention. The persistence of the observed benefits over time was not evaluated, and therefore the durability of the effect of mental imagery on PVC insertion skill retention remains to be determined.

Future research should address these issues by focusing on both immediate and long-term outcomes, assessing the sustainability of mental imagery effects on procedural performance over extended periods. Prior work has suggested that the benefits of mental practice may decline without reinforcement over time [23]. Given its ease of implementation and minimal resource requirements, mental imagery represents a particularly attractive strategy for promoting long-term retention, especially when integrated through repeated sessions over time. Investigating the optimal frequency and duration of mental imagery practice, including the potential additive effect of multiple sessions, therefore represents a key area for future studies in procedural training. While mental imagery is already well established in surgical education, its potential role in the acquisition and reinforcement of other invasive procedural skills, such as central venous catheterization, lumbar puncture, or endotracheal intubation, remains largely unexplored. Integrating mental practice into the training of these high-risk procedures could offer a valuable, low-cost adjunct to traditional simulation-based methods, with the potential to enhance both technical performance and safety outcomes across a wide range of clinical contexts.

## Conclusion

Mental imagery appears to be an effective, safe, and low-cost educational strategy, not only for enhancing the acquisition of procedural skills, but also for improving simulation-based performance.

Future studies should investigate the impact of mental imagery on other invasive procedures and non-procedural competencies and assess the sustainability of these benefits.

## Data Accessibility Statement

The dataset used and analyzed during the current study is available from the corresponding author on reasonable request.

## Additional File

The additional file for this article can be found as follows:

10.5334/pme.2069.s1 Supplementary Material. Figures S1, S2 and Table S1.
