# Supplementary material for: Mental Imagery to Enhance Procedural Skills in Peripheral Venous Catheterization: A Randomized Simulation Study in Medical Students
Source: Perspect Med Educ. 2026 Feb 18;15(1):176–84. doi: 10.5334/pme.2069 (PMC12922665; doi:10.5334/pme.2069)
Supplement: Supplementary Material. — Figures S1, S2 and Table S1. [file pme-15-1-2069-s1.pdf]

SUPPLEMENTARY MATERIAL - SUMMARY

Figure S1. Script for mental imagery instructions

Figure S2. Revised Mental Imagery Questionnaire-3 (MIQ)

Table S1. Detailed results of the revised MIQ-3 score

Figure S1. Script for mental imagery instructions

"When you picture the action, visualize all movements as parts of the action. To do this, close your eyes and try to clearly perceive all the steps involved in inserting the peripheral venous catheter, as if you were performing the movement. Do not move or imitate the movement. Position yourself in relation to the patient, as if you were going to perform the needle insertion

1. Disinfect your hands with hydro-alcoholic solution.
2. Put on non-sterile gloves.
3. Apply tourniquet.
4. Locate the vein to be punctured.
5. Disinfect the skin in 1 step, at the puncture site, using an antiseptic.
6. Spread and tighten the skin with your left hand. Locate the exact spot where you need to insert the needle.
7. Check the angle between needle and skin before insertion, which should be around 30 degrees.
8. Insert the needle into the patient's skin, bevel upwards, into the vein, exerting the appropriate force; try to feel the return of resistance from the tissues through which the needle passes.
9. Once in the vein lumen, stabilize the needle. Watch for blood flow into the catheter.
10. Slowly insert the catheter into the vein, sliding it along the needle.
11. Loosen the tourniquet.
12. Secure the catheter with the strip.
13. Remove the needle from the catheter, compressing the patient's vein with the other hand.
14. Connect the purged tubing to the catheter, and check that it is working.
15. Apply the transparent dressing.
16. Dispose of equipment, including blunt objects, in the appropriate contaminated waste container."

Translated from the French

Figure S2. Revised Mental Imagery Questionnaire (MIQ)-3

1. **How prepared do you feel to perform a peripheral venous catheter (PVC) insertion?**

Not at all ready	1	2	3	4	5	6	7	Very ready

2. **How confident do you feel about performing a PVC insertion?**

Not confident at all	1	2	3	4	5	6	7	Very confident

3. **To what degree do you think you can perform a PVC insertion compared to other people at your level?**

Impossible	1	2	3	4	5	6	7	Very easy

4. **To what degree did the activity you performed help you prepare for a PVC insertion?**

Not useful at all	1	2	3	4	5	6	7	Very useful

5. **How easily can you “see” yourself performing a PVC insertion?**

Impossible	1	2	3	4	5	6	7	Very easy

6. **How vivid and clear are the images of a PVC insertion in your mind?**

Not clear at all	1	2	3	4	5	6	7	Very clear

7. **How easy is it to “feel” you're doing a PVC insertion?**

Impossible	1	2	3	4	5	6	7	Very easy

8. **To what extent would you be able to describe the steps involved in inserting a PVC to someone?**

Impossible	1	2	3	4	5	6	7	Very easy

PVC: peripheral venous catheter

Table S1. Detailed results of the revised MIQ-3 score

S1a. Before the procedural workshop

**Characteristic**	**Mental Imagery** N = 33	**Control** N = 31	**Overall** N = 64
Question 1			
Median [Q1, Q3]	4 [3, 5]	5 [4, 5]	5 [3, 5]
Question 2			
Median [Q1, Q3]	4 [3, 5]	4 [3, 5]	4 [3, 5]
Question 3			
Median [Q1, Q3]	4 [3, 5]	4 [3, 5]	4 [3, 5]
Question 4			
Median [Q1, Q3]	1 [1, 1]	1 [1, 1]	1 [1, 1]
Question 5			
Median [Q1, Q3]	6 [4, 6]	5 [4, 5]	5 [4, 6]
Question 6			
Median [Q1, Q3]	5 [4, 5]	5 [4, 6]	5 [4, 6]
Question 7			
Median [Q1, Q3]	4 [3, 5]	4 [3, 5]	4 [3, 5]
Question 8			
Median [Q1, Q3]	4 [3, 5]	5 [3, 6]	4 [3, 5]

S1b. After the procedural workshop

Characteristic	**Mental Imagery**<br>N = 33	**Control**<br>N = 31	**Overall**<br>N = 64
Question 1			
Median [Q1, Q3]	6 [6, 7]	6 [5, 6]	6 [5, 7]
Question 2			
Median [Q1, Q3]	6 [5, 6]	6 [5, 6]	6 [5, 6]
Question 3			
Median [Q1, Q3]	5 [5, 6]	5 [4, 6]	5 [4, 6]
Question 4			
Median [Q1, Q3]	7 [6, 7]	6 [5, 7]	6 [5, 7]
Question 5			
Median [Q1, Q3]	6 [6, 7]	6 [5, 7]	6 [5.50, 7]
Question 6			
Median [Q1, Q3]	6 [6, 7]	6 [5, 7]	6 [6, 7]
Question 7			
Median [Q1, Q3]	6 [5, 7]	6 [5, 6]	6 [5, 6.5]
Question 8			
Median [Q1, Q3]	6 [5, 7]	6 [6, 7]	6 [5.5, 7]
